# A Quantitative Comparison of Cell-Type-Specific Microarray Gene Expression Profiling Methods in the Mouse Brain

**DOI:** 10.1371/journal.pone.0016493

**Published:** 2011-01-27

**Authors:** Benjamin W. Okaty, Ken Sugino, Sacha B. Nelson

**Affiliations:** Department of Biology, Brandeis University, Waltham, Massachusetts, United States of America; Max Planck Institute for Evolutionary Anthropology, Germany

## Abstract

Expression profiling of restricted neural populations using microarrays can facilitate neuronal classification and provide insight into the molecular bases of cellular phenotypes. Due to the formidable heterogeneity of intermixed cell types that make up the brain, isolating cell types prior to microarray processing poses steep technical challenges that have been met in various ways. These methodological differences have the potential to distort cell-type-specific gene expression profiles insofar as they may insufficiently filter out contaminating mRNAs or induce aberrant cellular responses not normally present *in vivo*. Thus we have compared the repeatability, susceptibility to contamination from off-target cell-types, and evidence for stress-responsive gene expression of five different purification methods - Laser Capture Microdissection (LCM), Translating Ribosome Affinity Purification (TRAP), Immunopanning (PAN), Fluorescence Activated Cell Sorting (FACS), and manual sorting of fluorescently labeled cells (Manual). We found that all methods obtained comparably high levels of repeatability, however, data from LCM and TRAP showed significantly higher levels of contamination than the other methods. While PAN samples showed higher activation of apoptosis-related, stress-related and immediate early genes, samples from FACS and Manual studies, which also require dissociated cells, did not. Given that TRAP targets actively translated mRNAs, whereas other methods target all transcribed mRNAs, observed differences may also reflect translational regulation.

## Introduction

Neurons differ widely in their morphology, firing properties, connectivity and other cellular phenotypes due in part to profound differences in gene expression. Recently, several approaches have been used to comprehensively measure cell-type-specific gene expression using microarrays. In each method, targeted cell types are identified visually, typically through expression of a fluorescent protein, but the methods differ in the way that targeted cells are separated from surrounding tissue. These differences may have important effects on the resulting data. For example, the degree to which a sample exclusively reflects expression in the targeted cells depends on the efficiency of the separation. On the other hand, vigorous dissociation of tissues associated with some separation methods can potentially cause activation of stress or cell death pathways thereby distorting the resulting expression profile. Here we quantitatively compare five different cell-type specific profiling approaches with respect to repeatability, accuracy, and sensitivity to several artifacts. Data from studies employing laser capture microdissection (LCM;[1], [2], translating ribosome affinity purification (TRAP;[3], [4], fluorescence activated cell sorting (FACS; [5]), immno panning (PAN; [6]), and manual sorting (Manual; [7], [8] to isolate cell-type-specific samples for hybridization with the same microarray platform (Affymetrix Mouse) were re-analyzed and directly compared. The results reveal that all methods are highly reproducible but that they differ in the apparent purity of the samples from contamination with transcripts from other cell types. Because some methods rely on dissociation while others do not, we also compared the degree of activation of apoptosis-related, stress-related and immediate early genes and found evidence for a mounted response in the PAN method, but not in the other two dissociation-based methods. Although most of the data was obtained from cell types not tested with more than one method, for a single cell-type, cerebellar Purkinje cells, it was possible to directly compare profiles obtained with three different methods: LCM, TRAP and Manual. Most known Purkinje cell markers were identified by all three approaches, but many other transcripts showed surprisingly large differences. Some of these differences may reflect differential contamination, while others may reflect real differences in the degree to which transcripts are actively translated.

## Methods

### Cell-type-specific purification methods

Details of the methods used in each of the reviewed studies (LCM, TRAP, PAN, FACS, and Manual) can be found in the corresponding references given in the introduction. Note, the Cahoy, et al. 2008 study from which the PAN data was obtained employed a combination of PAN and FACS to varying degrees, however given that all purified cell types from this study were initially subject to panning purification, we refer to the method simply as PAN. All studies used similar methods for isolation and cleanup of RNA from either isolated cells (LCM, PAN, FACS, and Manual) or from immunoprecipitated polysomes. Also, all studies used two round T7-mediated In Vitro Transcription (IVT) for amplification and labeling of RNA and used similar input amounts of cRNA for microarray hybridization.

### Analysis of microarray data

We obtained microarray data (CEL files) from the Gene Expression Omnibus (GEO) or directly from the authors of six published studies as well as from one unpublished study (Table 1, Table S1).This included all cell type-specific microarray data available at the outset of the analysis which made use of the Mouse430v2 or MOE430A Affymetrix platform (only probes common to both platforms were used). All the CEL files were subjected together to background correction, normalization and summary value calculation using Affymetrix Power Tools (apt version 1.8.6 using “rma-sketch” option) (http://www.affymetrix.com/partners_programs/programs/developer/tools/powertools.affx). Resulting summary values were used for the analysis.

**Table 1:** An overview of the different purification methods, references, number of cell types, and number of microarrays used in the analysis.

Method	References	# Cell Types	# Microarrays
LCM	Chung et al., 2005; Rossner et al., 2006	5	19
TRAP	Doyle et al., 2008; Heiman et al., 2008	24	76
FACS	Arlotta et al., 2005	7	15
PAN/FACS	Cahoy et al., 2008	8	23
Manual	Sugino et al., 2006; Okaty et al., 2009	20	62
	**Total**	64	195

### Calculating replicate correlation coefficients

We treated each of the 195 samples as a 22,690-element vector, where each element corresponds to the log 2 normalized microarray signal-intensity for a given probe set on the Affymetrix Mouse 430 A gene chip. We then computed the Pearson product-moment correlation coefficients between all pairings of biological replicate samples for each of the 64 profiled cell types.

### Contamination Indices

The procedure for calculating the contamination index of a given sample was as follows. Non-GABAergic sample microarray signal values for a given GABAergic marker gene (Slc32a1 (Vesicular GABA transporter), Gad1, Gad2) were normalized to the range [0,1], where a value of 0 corresponded to the lowest expression level of that gene among the non-GABAergic samples and a value of 1 corresponded to the maximum expression level of that gene among the GABAergic samples (Figure S1a). We then obtained the GABAergic contamination index of each non-GABAergic sample by averaging over the normalized signal values of multiple marker genes. In the same manner, we obtained the astrocyte (marker genes: Aqp4, Gfap, Fgfr3, Slc1a2, Gjb6) and oligodendrocyte (marker genes: Mbp, Sox10, Mag, Mog) contamination indices (Figure S1b,c). A second index was computed for each contamination category using an expanded set of genes selected by clustering analyses. Focusing on the most significantly differentially expressed genes (minimum of 10-fold difference in expression between at least 2 cell types and an ANOVA p-val < 1e-70; a total of 1612 genes) we first performed hierarchical clustering of genes across samples and then of samples across genes, using the Euclidean distance metric and average-linkage (unweighted pair-group method average), and then plotted a heat map of expression levels for these 1612 genes for each cell type, where the genes (rows) and cell types (columns) are ordered by their corresponding hierarchical clusters. The results of these analyses are given in Figure S2. For astrocyte and oligoendendrocyte samples, large blocks of highly enriched genes can be readily discerned. We selected the “best-looking” subset (i.e. uniformly high across cells of a similar type, and low otherwise) of these clustered genes for each cell type to compute the expanded contamination index (42 genes for astrocytes and 26 genes for oligodendrocytes; Figures S3 and S4). GABA gene clusters were less apparent and consisted of fewer genes, perhaps reflecting the greater heterogeneity between different subsets of GABAergic neurons. This poorer clustering led us to relax the significance threshold (ANOVA p value < 1e-40) and to include markers of interneuron subsets, such as Sst and Lhx6, rather than strictly pan-GABAergic genes (12 genes in all; Figure S5). It should be noted that the greatest “improvements” in purity are seen for GABAergic contamination with this second index, which may be a direct consequence of including less general GABAergic markers.

### Lists of genes for assessing expression artifacts

We obtained a list of Immediate Early genes (IEGs) from [9], the apoptosis gene set was constructed using the WhichGenes gene set building tool (http://www.whichgenes.org/), and stress genes were selected on the basis of Gene Ontology (GO) annotation (Table S3).

### Determination of glia-enriched genes for filtering out probable contaminants

A non-glial comparison group was constructed by combining all samples with lower than 0.2 astrocyte and oligodendrocyte contamination indices as calculated above, and t-tests were performed between these samples and the astrocyte and oligodendrocyte sample groups for all genes. Genes with a t-test p-value <0.001 and showing a more than 2-fold enrichment in the glial samples were considered glia-enriched, and thus may reflect glial contamination when expressed in non-glial profiles (Table S5a,b).

### Analysis of UTR and ORF sequence length

We downloaded full length mRNA and protein sequences for all mouse genes (http://www.ncbi.nlm.nih.gov/Ftp/), and calculated UTR lengths by subtracting three times the protein sequence length from the length of the full mRNA for each gene represented on the MOE 430 A chip for which we were able to map the affymetrix identifier to the RefSeq identifier (∼10,000 genes).

### Determination of Purkinje enriched gene

T-tests were performed between all Purkinje samples taken as a single group and all Non-Purkinje samples taken as a group for all genes. Heirarchical clustering was performed as described above. Genes included in Figure S9 are tightly clustered, have a t-test p-value < 1e-20 and show >10-fold difference in expression between group means.

## Results

We compared microarray data from 7 different studies, utilizing 5 different cell-type-specific mRNA isolation methods, and representing a total of 64 different cell types (Table 1; Table S1). The 5 methods were: LCM, TRAP, PAN, FACS, and Manual.

### Repeatability

**Figure 1:**
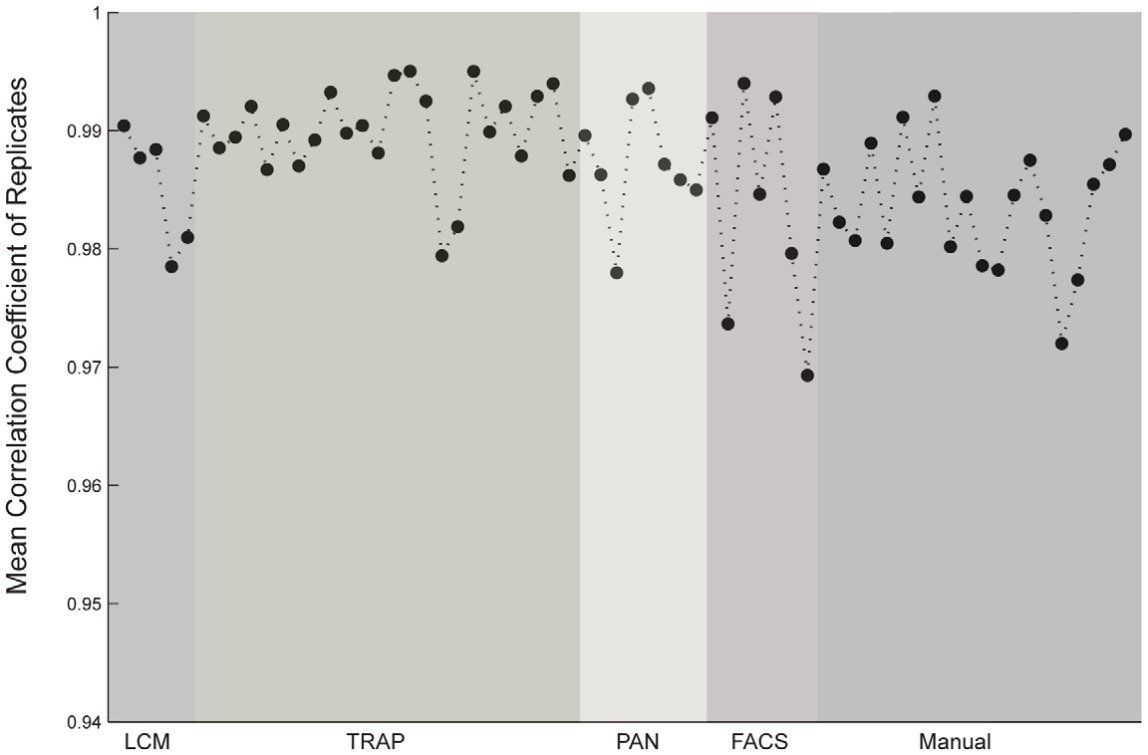
Repeatability of microarray measurements is high and largely uniform across all cell purification methods. Each data point represents the mean Pearson product-moment correlation coefficient between biological replicates for each of the analyzed cell types. Cell types are grouped by purification method, corresponding to the labels on the horizontal axis and demarcated by the shaded regions.

In order to assess the repeatability of purification methods, we first computed the mean correlation coefficient between biological replicates for each cell type (Figure 1; see Methods). Then we performed a one-way ANOVA across methods, followed by Tukey's post hoc test, and found an extremely modest but significant difference between the repeatability of the TRAP and Manual methods (ANOVA p-val <0.01, Tukey's post hoc test p-val <.0.05), with the TRAP replicate samples showing a 1.006-fold higher correlation on average than the Manual samples. Given that gene regulation is a stochastic process which can lead to significant cell-to-cell variability in the expression levels of various transcripts even within a discrete class of cells [10], [11], [12], [13], this result was not altogether unexpected insofar as these two methods represent the maximum (>10,000) and minimum (50-100) number of cells, respectively, from which mRNA was extracted among all of the compared methods. However, given the very small magnitude of the effect, it also suggests that cell-to-cell variability among well-defined neural cell types may likewise be relatively small.

### Sample Purity and Contamination

**Figure 2:**
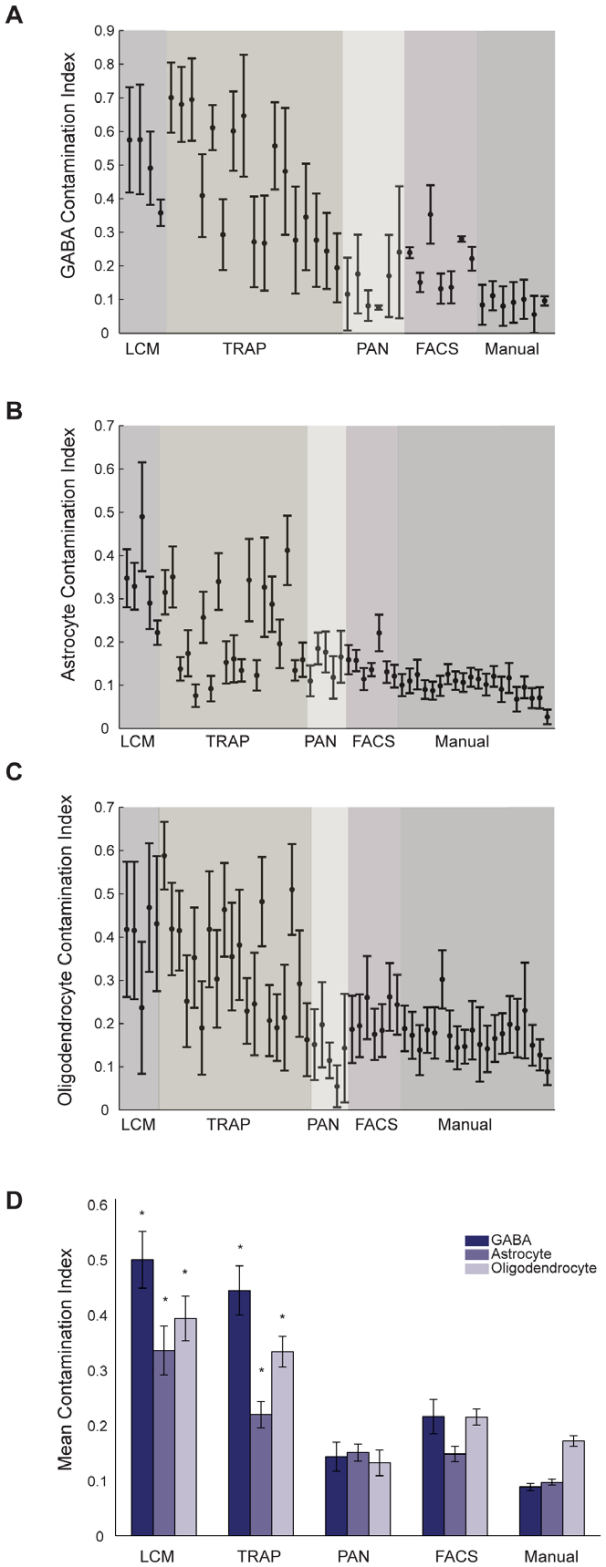
Cell purification methods show differential susceptibility to contamination. (**A**) GABA contamination indices of non-GABAergic cell types, (**B**) astrocyte contamination indices of non-astrocyte cell types, and (**C**) oligodendrocyte contamination indices of non-oligodendrocyte cell types. For an explanation of how the contamination indices were computed, see the *Methods*. (**D**) Mean sample contamination indices of each purification method for the three different categories of contaminants (from **A**, **B**, and **C**). Differences in mean contamination indices across methods were significant for each category of contaminant (ANOVA p<0.005). Asterisks indicate which means were significantly different from the lowest means (Tukey's post-hoc test, p<0.05), which were achieved by the Manual sorting method in the case of GABA and astrocyte contamination, and PAN in the case of oligodendrocyte contamination.

As a first step toward assessing sample purity, we partitioned the samples into six gross categories: GABAergic neurons, non-GABAergic neurons, astrocytes, non-astrocytes, oligodendrocytes, and non-oligodendrocytes (Table S2a,b, and c). Our selection of these categories was motivated by the fact that well-recognized cell-type-specific marker genes for GABAergic cells, astrocytes, and oligodendrocytes were reliably represented by probes on the Affymetrix Mouse 430 A gene chip. In order to quantify the degree to which, for example, the non-GABAergic cell samples contained contaminating GABAergic cell-specific mRNAs, we computed a contamination index for each of the non-GABAergic samples, and likewise for the other two categories of contamination (see Methods; Figure 2 a,b,c). We then performed ANOVAs across methods for each contamination category, followed by Tukey's post-hoc test, and found that the mean contamination indices for LCM and TRAP were significantly higher than PAN, FACS, and Manual for GABA and oligodendrocyte contamination (Figure 2d; GABA: ANOVA p-val < 1e-6, Tukey's post hoc p-val <0.05, maximum fold difference between methods ∼5; Oligodendrocyte: ANOVA p-val < 1e-7, Tukey's post hoc <0.05, maximum fold difference between methods ∼3.4), and that the mean LCM astrocyte contamination index was significantly higher than all other methods and the TRAP astrocyte contamination was significantly higher than Manual (ANOVA p-val < 1e-7, Tukey's post hoc p-val <0.05, maximum fold difference between methods ∼2.9).

While these indices were based on the expression levels of undisputed cell-type-specific marker genes, a possible shortcoming is that they were calculated using a relatively small number of genes (3 for GABA, 5 for astrocytes, and 4 for oligodendrocytes). Thus we computed alternative contamination indices using an expanded set of genes selected by unsupervised clustering of gene expression profiles across cell types (see Methods). The Pearson correlation coefficient of the original contamination index and this second index was .81, p-val < 1e-4 (Figure S6A). While on average the values of the second index were lower than the first, some samples showed a higher value, and except in the case of PAN and FACS glial contamination the ordinal relationships between methods were maintained (i.e. the mean contamination index for LCM samples was always highest and Manual was always lowest, etc.; Figures S6B,C, and D). The contamination indices for each sample can be found in Tables S2a,b, and c.

### Expression Artifacts

**Figure 3:**
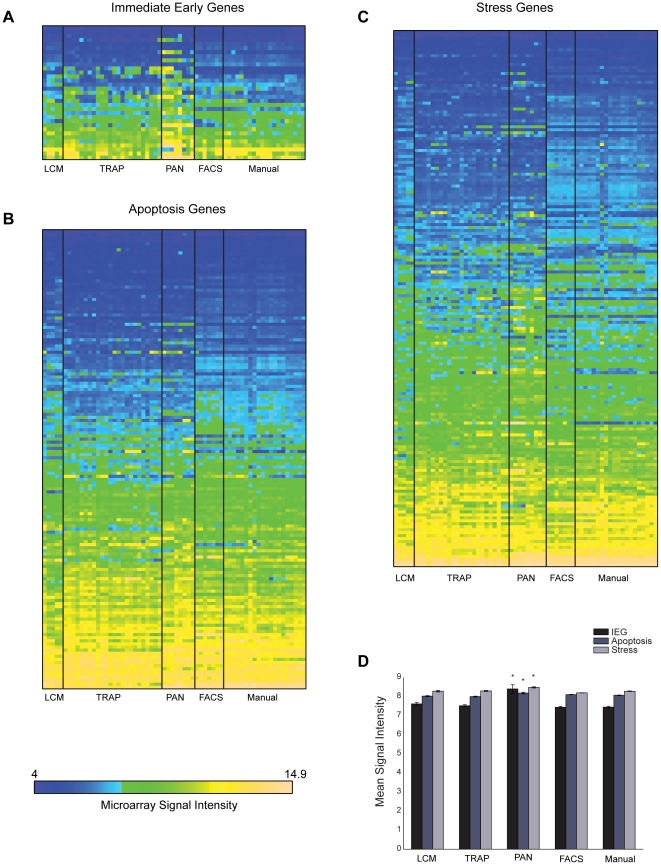
PAN samples show moderately heightened expression of (A) immediate early genes, (B) apoptosis-related genes, and (C) stress-responsive genes, however overall global trends are comparable for most samples. For each heat map, the replicate-averaged log2 microarray signal intensity (normalized) for each cell type (columns) is presented for all genes (rows) in a given category. Horizontal axis labels and vertical lines indicate purification method groups. (D) Average mean signal intensity for each method and each gene category. Asterisks indicate which means were significantly different from the lowest means (Tukey's post-hoc test, p<0.05).

The separation of select populations of cells from acutely dissected tissue poses several technical challenges. For example, potential stressors introduced in the intervening steps between tissue extraction and mRNA isolation may distort the resulting portrait of cell-type-specific transcriptional state if they induce a transcriptional response not representative of the in vivo state. Potential stressors include antibodies, enzymes and other reagents used for immunopanning or to digest tissue, non-physiological variations in temperature and other aspects of the cellular environment, as well as the mechanical stress of physically breaking up the tissue. Since the longer it takes to extract the mRNA, the greater the likelihood of a transcriptional response, the reliability of methods requiring cellular dissociation, such as PAN, FACS, and Manual, may be more prone to these effects than methods that do not require dissociation, such as LCM and TRAP. In order to investigate the prevalence of these speculative effects, we focused on three categories of gene function – immediate early genes (IEGs), apoptosis related genes, and stress related genes (see Methods). Heat maps depicting the expression levels of genes for each of the three categories show similar global trends across samples and methods (Figure 3a,b,c), however there are many clear cases of cell-type and method differences. In order to quantify the differences in the overall effects for each method, we computed the mean values for each sample over all genes within a category, and then performed an ANOVA on the sample means across methods for each category. By this measure, PAN samples showed significantly higher levels of expression of IEGs, apoptosis related genes, and stress related genes than other methods (IEG: ANOVA p-value < 1e-8, Tukey's post hoc p-value <0.05, maximum fold difference between methods >∼1.9; Apoptosis: ANOVA p-value <0.001, Tukey's post hoc p-value <0.05, maximum fold difference between methods >∼1.1; Stress: ANOVA p-value <1 e-5, Tukey's post hoc p-value <0.05, maximum fold difference between methods >∼1.2). However, compared to the magnitude of the contamination effects, heightened expression of these categories of genes in PAN samples was relatively modest. The comparison also revealed a lack of any significant difference between LCM and TRAP versus FACS and Manual, suggesting that dissociation in and of itself does not necessarily induce an appreciable stress response.

It is important to note that given the largely non-overlapping array of cell types profiled by each method, a possible confound of the foregoing analysis is that intrinsic differences in the amenability to purification of specific cell types may contribute to purported method effects. Moreover, several IEGs, apoptosis-related genes, and stress-related genes appear to be differentially expressed between different cell types. For example, the bulk of the PAN samples are from astrocytes and oligodendrocytes, and a subset of the genes elevated in PAN appear to be likewise elevated in TRAP astrocyte and oligodendrocyte samples. However, data from at least two methods was available for five cell types: cortical pyramidal neurons (TRAP,LCM, and Manual data), cerebellar Purkinje neurons (TRAP,LCM, and Manual data), cortical astrocytes (TRAP and PAN data), cortical oligodendrocytes (TRAP and PAN data), and cortical GABAergic interneurons (TRAP and Manual). In each of these cases LCM or TRAP showed the highest levels of contamination and Manual or PAN showed the lowest levels of contamination as measured by the two contamination indices (Table S2d).

### Within Cell-Type Comparison

**Figure 4:**
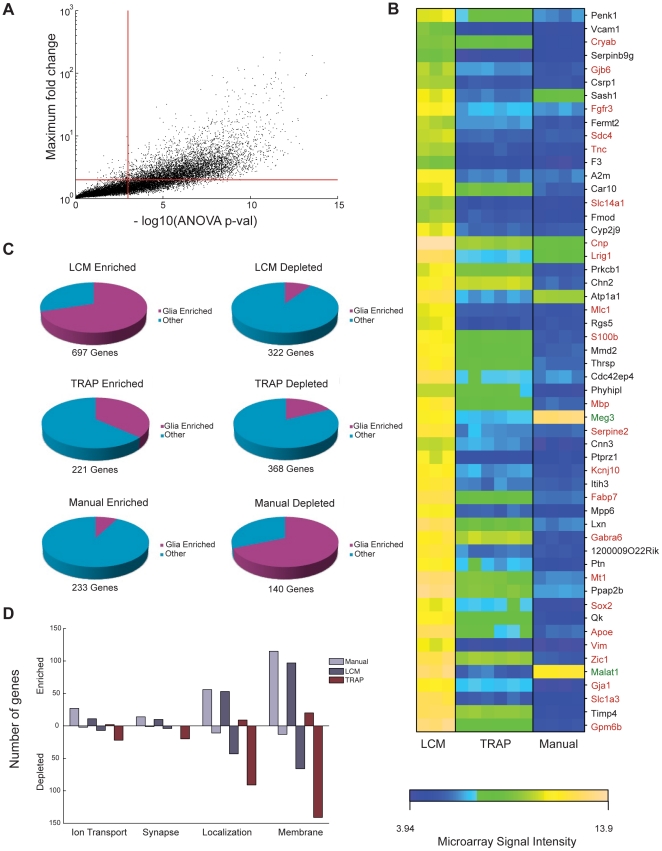
Gene expression profiles of Purkinje cells purified by three different methods show striking differences. (**A**) Scatter plot depicting the maximum fold difference of expression level between methods (vertical axis) and the corresponding ANOVA p-value (horizontal axis) for all genes (where an ANOVA was performed for each gene across groups defined by purification method). Upper right quadrant formed by the two intersecting red lines delineates significantly differentially expressed genes (maximum fold difference >2, ANOVA p-value < 1e-3). (**B**) Heat map depicting the most significantly differentially expressed genes (maximum fold difference between methods >20, ANOVA p-value < 1e-10) where each column represents an individual replicate sample. Gene names given in red font indicate genes that have a strong likelihood of being non-Purkinje gene contaminants (based on Allen Brain Atlas *In-situ* data and literature searches, see *Results*), green font indicates non-translated mRNAs, and the expression specificity of the remaining genes is unknown. (**C**) Pie graphs depicting the percentage of method enriched and depleted genes that are also enriched in glia, and thus are likely to be the result of contamination. The total number of genes in each category is given beneath each graph. **(D)** Gene ontology terms associated with Manual and LCM enriched genes and TRAP depleted genes. Number of genes associated with each term is given on the vertical axis.

Ideally, a comparison between methods should be conducted using identical cell types. While different means of cellular identification were employed in each case, cerebellar Purkinje cell profiles were common to LCM, TRAP, and Manual studies. Whereas Purkinje cells were identified by virtue of anatomy and morphology alone in the LCM study, restricted expression of a GFP transgene [14] aided the purification of Purkinje cells in the Manual method, and pull down of EGFP-ribosomal fusion protein allowed for the purification of Purkinje cell mRNA in TRAP. Each method successfully detects the majority of known Purkinje cell markers [15]; Figure S7), however, we found that 5,314 genes were differentially expressed between LCM, TRAP, and Manual, using a criterion of <1e-3 ANOVA p-value and a minimum fold-change of 2 (Figure 4a). As a first pass at characterizing the types of genes that were differentially expressed, we first focused on the most significant, namely those genes with less than a 1e-10 ANOVA p-value and showing a greater than 20-fold difference in expression between methods (Figure 4b). 55 genes met this criterion. By combining literature searches with Allen brain atlas *in situ* hybridization data, we established that 23 of these genes were likely the result of contamination from non-Purkinje cells (Figure 4b, gene names in red font; Table S4). 17 of these genes showed the highest signal level in the LCM samples, with intermediate to low levels in TRAP, and the lowest levels in Manual. A handful of genes appeared to be non-translated RNAs which showed low signal in the TRAP profiles (Figure 4b, gene names in green font), reflecting the fact that TRAP only targets RNAs associated with ribosomes, and hence only those RNAs which are actively being translated at the time the tissue is processed.

To further characterize genes that were differentially expressed as a result of method, we performed a series of t-tests in which the Purkinje samples from a given method were compared against samples from the other two methods combined, and examined those genes with a p-value less than 0.001 and a fold-difference >3. This allowed us to distinguish between genes that were significantly enriched or depleted in each method. Given the high number of genes enriched and depleted in each method, we needed a systematic way of assigning differentially expressed genes to informative categories. Thus we applied GO overrepresentation analysis to enriched and depleted sets of genes, as well as our own filter for glia-enriched contaminant genes (see Methods). Taking the intersection of each set of method-enriched and depleted genes with the glia-enriched gene set, we found that roughly 70% of the LCM Purkinje enriched genes are also glia-enriched, versus 37%, and 8% respectively for TRAP and Manual. Reciprocally, 69% of the genes that are depleted in Manual with respect to the other two methods are glia-enriched (Figure 4c). This pattern of contamination is consistent with the contamination indices computed previously (Figure 2b,c; LCM Purkinje mean contamination index: 0.36, TRAP Purkinje mean contamination index: 0.16, Manual Purkinje mean contamination index: 0.10).

In order to focus on method differences not overtly related to glial contamination, we restricted GO overrepresentation analysis to non glia-enriched genes, and found that the remaining method enriched and depleted genes showed significant enrichment for several categories of gene function and cellular localization (Table S6a,b,c). Interestingly, LCM and Manual enriched and depleted genes are associated with many of the same gene ontology terms. For example, genes associated with the biological processes of “ion transport” and “localization” are overrepresented among LCM and Manual enriched genes, and thereby overrepresented in TRAP depleted genes (Figure 4d). Likewise genes localized to the synapse and membrane are enriched in LCM and Manual Purkinje, but depleted in TRAP (Figure 4d).

Given that LCM and Manual methods profile all transcribed mRNAs, whereas the TRAP method only profiles mRNAs associated with tagged ribosomes, lower expression of specific genes in TRAP data may reflect lower ribosome density and/or ribosomal occupancy of these transcripts, resulting in translational suppression [16], [17], [18]. One mechanism by which mRNAs are post-transcriptionally regulated is through interactions with RNA binding proteins and sequences contained in their 3′ and 5′ untranslated regions (UTRs) [19]. Given that longer UTRs in theory have a higher probability of containing regulatory sequences [20], we looked for correlations between suppressed expression in the TRAP data and UTR length (see Methods). We found that the mean UTR length of TRAP depleted genes is 1.3-fold higher than the mean UTR length of all MOE 430 A chip transcripts (t-test p-value  = 8.27e-5). However we found no significant correlation between the UTR length of TRAP depleted transcripts and the magnitude of suppressed expression as measured by the fold change in expression between TRAP and the other methods. To our surprise, we also found that the mean length of the coding sequences, or open reading frames (ORFs), of TRAP depleted genes is 2.02-fold greater than the mean ORF length of all MOE 430 A chip transcripts, and that the ORF length of TRAP depleted genes shows a modest but significant correlation with fold-suppression (Pearson product-moment correlation coefficient  = 0.28, p-val < 1e-4). These results are summarized in Figure S8.

Interestingly, comparison between TRAP profiles and profiles obtained from other methods for cortical pyramidal neurons, cortical astrocytes, cortical oligodendrocytes, and cortical GABAergic interneurons all showed similar trends. The mean ORF lengths of TRAP depleted genes ranged from 1.38-fold (cortical oligodendrocytes) to 2.04-fold (cortical GABAergic interneurons) higher than the average ORF length for all MOE 430 A genes. Also, the GO cellular component term “plasma membrane” is overrepresented in the set of TRAP depleted genes for each of these cell types.

Despite possible differences in the translational efficiency of particular subsets of genes, differential contamination, or other potential artifacts, concordant microarray data across Purkinje samples obtained from these diverse methods provides stronger evidence for genuine expression than from any one study alone. Moreover, the vast number of cell types compiled for this study affords a highly inclusive comparison group for identifying cell-type enriched genes. Thus we used both t-tests and clustering to identify Purkinje enriched genes (see Methods). The results of these analyses corroborated known Purkinje enriched genes and also identified novel marker genes, such as Nrk, Ebf1, Smpx, Il22/Iltitfb (single probe set covers sequences common to both genes), and Krt25. The full set of enriched genes can be found in Figure S9.

## Discussion

Faithful representation of the *in vivo* global transcriptional or translational state of a given class of neural cells using microarrays is encumbered by the underlying structure of brain tissue: heterogeneous, spatially intermingled cell types, distributed in varying proportions. An ideal method for purifying cell-type-specific mRNAs must thereby optimize selectivity for one class of cells above all others while minimizing the potential for artificially perturbing gene expression in the process. We compared microarray data from five different methods and found significant differences in the extent of contamination and stress artifacts. LCM and TRAP data showed significantly higher levels of contamination than the other methods, and PAN data showed elevated levels of IEGs and apoptosis and stress related genes. Given the high cell density of brain tissue, contamination in the case of LCM may result from the technical difficulty of restricting the microdissecting laser to the contours of only a single cell body, thus allowing closely apposed cells to be dissected along with the cell of interest. Contamination of TRAP samples may be a consequence of pulling down tagged ribosomes from tissue homogenate, which contains mRNA from numerous non-target cell types. PAN, FACS, and Manual all sort target cells from a population of mostly intact dissociated cells, thus the risk of contamination from mRNA in the surrounding medium is smaller. Alternatively, contamination in the TRAP samples could reflect low level expression of the EGFP-L10a transgene outside of the intended population. For example, the Etv1 BAC line used in the Doyle, et al. 2008 TRAP study labels predominantly Layer 5a corticostriatal pyramidal neurons, however EGFP expression in this line can also be faintly detected in astrocytes, which is in turn reflected in the expression profile of these samples (data not shown). We did not however include any such lines, for which off-target transgene expression was explicitly known, in our analysis of contamination. Transgene expression in mixed cell populations is a common feature of transgenic mouse lines, as single genes are often insufficient to fully delineate a single cell type. In these cases, PAN and Manual may allow a greater degree of selectivity than the other methods, insofar as the expression of surface proteins may serve as an additional filter in the case of PAN, and cell morphological features may further inform selection of target cells in the Manual method. However the long processing time required for performing antibody reactions in PAN may induce aberrant gene expression in target cells as a result of prolonged exposure to an artificial environment.

Both contamination and stress artifacts are somewhat variable between within-method samples, however, suggesting that the purification of certain cell types may be more tractable than others. Interpretation of the stress effects is further complicated by the fact that some of the observed gene expression differences may be the result of intrinsic cell-type differences rather than method differences. Also, PAN and FACS data came from early postnatal mice whereas the majority of the data from the other studies came from mature mice (Table S1). Thus differential contamination and stress effects in these cases may in part reflect developmental differences rather than method differences. With these caveats in mind, we chose to focus our analyses more closely on Purkinje cell data, for which LCM, TRAP, and Manual data was available. We detected thousands of differentially expressed genes between the three methods. Given that non-biological “batch effects” are a common occurrence in microarray experiments [21], some amount of variance is to be expected when comparing different batches of microarray data. Sources of this variation may include differences in the efficacy of amplification reagents, the use of different RNA isolation kits, different atmospheric pressure and temperature conditions, and different locations. While each method successfully identifies Purkinje cell-specific marker genes, in theory differences in the sensitivities of each method may also result in differential expression of transcripts. However, we found that some of the observed differences in gene expression level could be accounted for by differential contamination. Consistent with the results of our analyses using all samples, we detected the highest degree of glial contamination in the LCM Purkinje data, with intermediate levels in TRAP data, and the lowest in Manual. Gene Ontology overrepresentation analysis suggests that many interesting categories of gene function and cellular compartmentalization, such as “ion transport” and “synaptic localization”, are commonly enriched in LCM and Manual data with respect to TRAP data. An important distinction of the TRAP method is that it selectively pulls down polysomal mRNAs, and therefore targets only translationally active mRNAs. This can be seen most clearly in the low or absent signal levels of non-coding RNA probes in TRAP data versus the other methods. Thus, excluding method artifacts, genes showing consistent expression in LCM and Manual, but diminished expression in TRAP may indicate translational suppression. Consistent with this hypothesis, mounting evidence suggests that many mRNAs encoding dendritic and synaptic proteins [19], [22], [23], as well as proteins enriched in other cellular compartments, are trafficked to and ultimately translated at the site of their localization, rather than in the cell soma. Through a variety of mechanisms, these trafficked mRNAs are often translationally repressed en route to their destination and their translation is often dependant on activity or signaling, and hence one would expect lower signal values for these transcripts in TRAP data, as indicated by GO overrepresentation analysis. In principle, regardless of the intervening regulatory mechanisms, the translational efficiency of a given gene is primarily influenced by its ribosomal occupancy, or the fraction of the encoding mRNA molecules associated with at least one ribosome, and the ribosome density, which is the number of ribosomes associated with that transcript per unit length. Thus translational suppression may reflect a decrease in either or both of these factors. Additionally, a number of studies have demonstrated a negative correlation between ribosome density and ORF length, the reasons for which remain enigmatic [16], [24], [25]. The observation that the mean ORF length of TRAP depleted genes is greater than the average ORF length for all genes, and that there exists a moderate correlation between the degree of reduced TRAP signal and ORF length, suggests that the TRAP signal in part reflects ribosome density, and not simply ribosome occupancy as might be expected.

Thus systematic comparison of TRAP data with data obtained by transcriptional profiling methods for a variety of cell types may facilitate a global perspective on cell-type-specific post-transcriptional regulation. However, it is important to note that the ability to detect differentially regulated transcripts within a particular cell type by comparing data obtained by different methods may be impeded by artifacts associated with a given method. Likewise, attempts at inferring the structure of intracellular gene networks by comparing microarray data across different cell types is potentially confounded by the inclusion of contaminating mRNAs, insofar as they distort the true set of interacting genes [26], [27]. Our analyses, together with other analytical approaches that have been developed to identify cell-specific genes from contaminated data [28], suggest ways of detecting and ultimately filtering out contamination artifacts in order to hone in on meaningful expression differences. This is especially important in the case of LCM, which by our analyses shows the greatest propensity for contamination, however is a uniquely indispensable tool in isolating cell types of interest from human post-mortem tissue [29], [30], [31]. Ultimately, despite differences in sample purity or other method artifacts, comparison across datasets from multiple studies and cell types has the potential to yield insights beyond that of any single study. As one example, our lists of astrocyte, oligodendrocyte, and Purkinje enriched genes (Tables S5a,b; Figure S9) were derived from comparisons between expression profiles from the largest number of cell types assembled to date, and are thus novel and valuable resources for applications in which expression “signatures” of these cell types are desired. Understanding how method differences influence the resulting data will aid future efforts to mine and analyze combined expression data, if not lead to improvements in the methods themselves.

## Supporting Information

Figure S1 **Normalized signal intensities of known (A) GABAergic,(B) astrocyte, and (C) oligodendrocyte and marker genes.**
(TIF)

Figure S2 **Dendrograms and heat map of highly significantly differentially expressed genes (see** **Methods** **).** Genes (rows) and cell types (columns) were clustered using Euclidean distance metric and average linkage. Microarray signal intensity values were standardized (across rows) such that the mean (i.e. mean signal value of a given gene across all samples) is zero and the standard deviation is one. Notice the primary division of glia and neurons.
(TIF)

Figure S3 **Normalized signal intensities of astrocyte enriched genes selected by clustering.** Columns are sorted by method.
(TIF)

Figure S4 **Normalized signal intensities of oligodendrocyte enriched genes selected by clustering.** Columns are sorted by method.
(TIF)

Figure S5 **Normalized signal intensities of GABAergic neuron enriched genes selected by clustering.** Columns are sorted by method.
(TIF)

Figure S6 **Comparison of two different contamination indices.** The first was calculated based on the expression of well established marker genes alone, whereas the second was based on the expanded sets of genes selected by clustering (Figures S3, S4, S5). (**A**) Scatter plot of the two different contamination indices (correlation  = .81). Comparison of the mean contamination indices for each method for (**B**) GABA contamination, (**C**) astrocyte contamination, and (**D**) oligodendrocyte contamination.
(TIF)

Figure S7 **Purkinje samples from all three methods identify most known marker genes.** Microarray signal levels are represented as a heat map for all cerebellar samples.
(TIF)

Figure S8 **The mean UTR and ORF lengths of TRAP depleted genes are significantly higher than the mean UTR and ORF lengths for all annotated genes on the MOE 430 A gene chip, and show modest but significant correlation with the degree of suppressed expression.** (**A**) Histogram depicting the normalized frequency of ORF lengths for all genes and for only TRAP depleted. (**B**) Histogram depicting the normalized frequency of UTR lengths for all genes and for only TRAP depleted.
(TIF)

Figure S9 **Heat map of purkinje enriched genes.**
(TIF)

Table S1 **A brief description of each analyzed cell type, and the study from which it came, including anatomical region, cell type, mouse age, RNA isolation and amplification method, microarray input RNA amount, and microarray platform.**
(XLS)

Table S2 **Sample groups used for computing contamination indices and the values of the contamination indices.** (**A**) Non GABAergic and GABAergic samples. (**B**) Non astrocyte and astrocyte samples. (**C**) Non oligodendrocyte and oligodendrocyte samples (D) Comparison of contamination indices for groups of samples representing similar cell types. Maximum and minimum values are indicated by yellow or blue shading, respectively.
(XLS)

Table S3 **Immediate Early Genes, Apoptosis Genes, and Stress genes (see** **Methods** **).**
(XLS)

Table S4 **Suspected non-Purkinje contamination genes.** Links to Allen Brain Atlas *in situ* hybridization data and PubMed identifiers of references indicating non-Purkinje expression of the corresponding gene.
(XLS)

Table S5 **Glia-enriched genes.** (**A**) Astrocyte enriched genes and the corresponding fold enrichment, and t-test p-value for each gene. (**B**) Oligodendrocyte enriched genes and the corresponding fold enrichment, and t-test p-value for each gene.
(XLS)

Table S6 **GO overrepresentation analysis for Purkinje Cells.** (**A**) LCM enriched and depleted, (**B**) TRAP enriched and depleted, (**C**) Manual enriched and depleted.
(XLS)
