# Supplementary figures and images for: A Quantitative Comparison of Cell-Type-Specific Microarray Gene Expression Profiling Methods in the Mouse Brain
Source: PLoS One. 2011 Jan 27;6(1):e16493. doi: 10.1371/journal.pone.0016493 (PMC3029380; doi:10.1371/journal.pone.0016493)

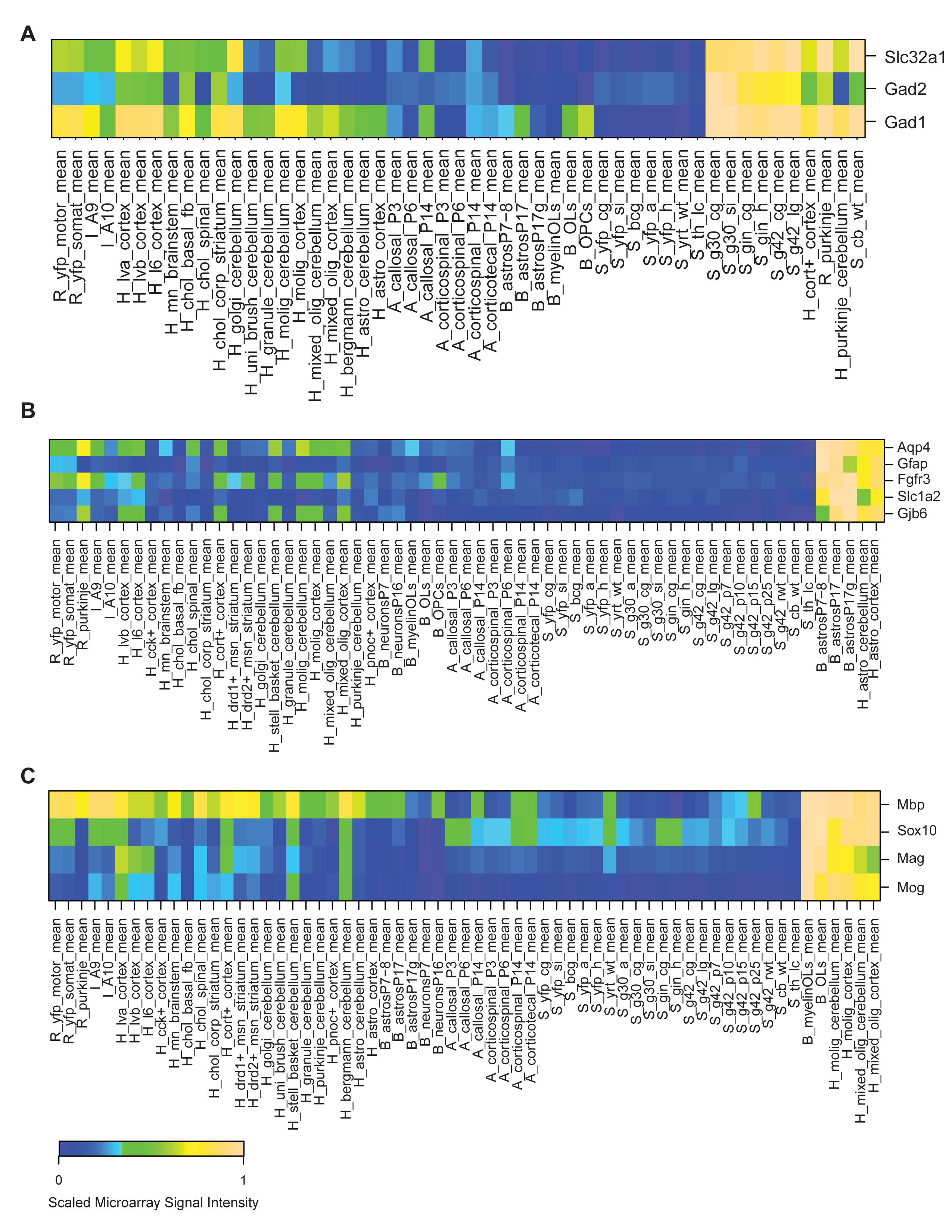

Supplement: Figure S1 — Normalized signal intensities of known (A) GABAergic,(B) astrocyte, and (C) oligodendrocyte and marker genes. (TIF) [file pone.0016493.s001.tif]

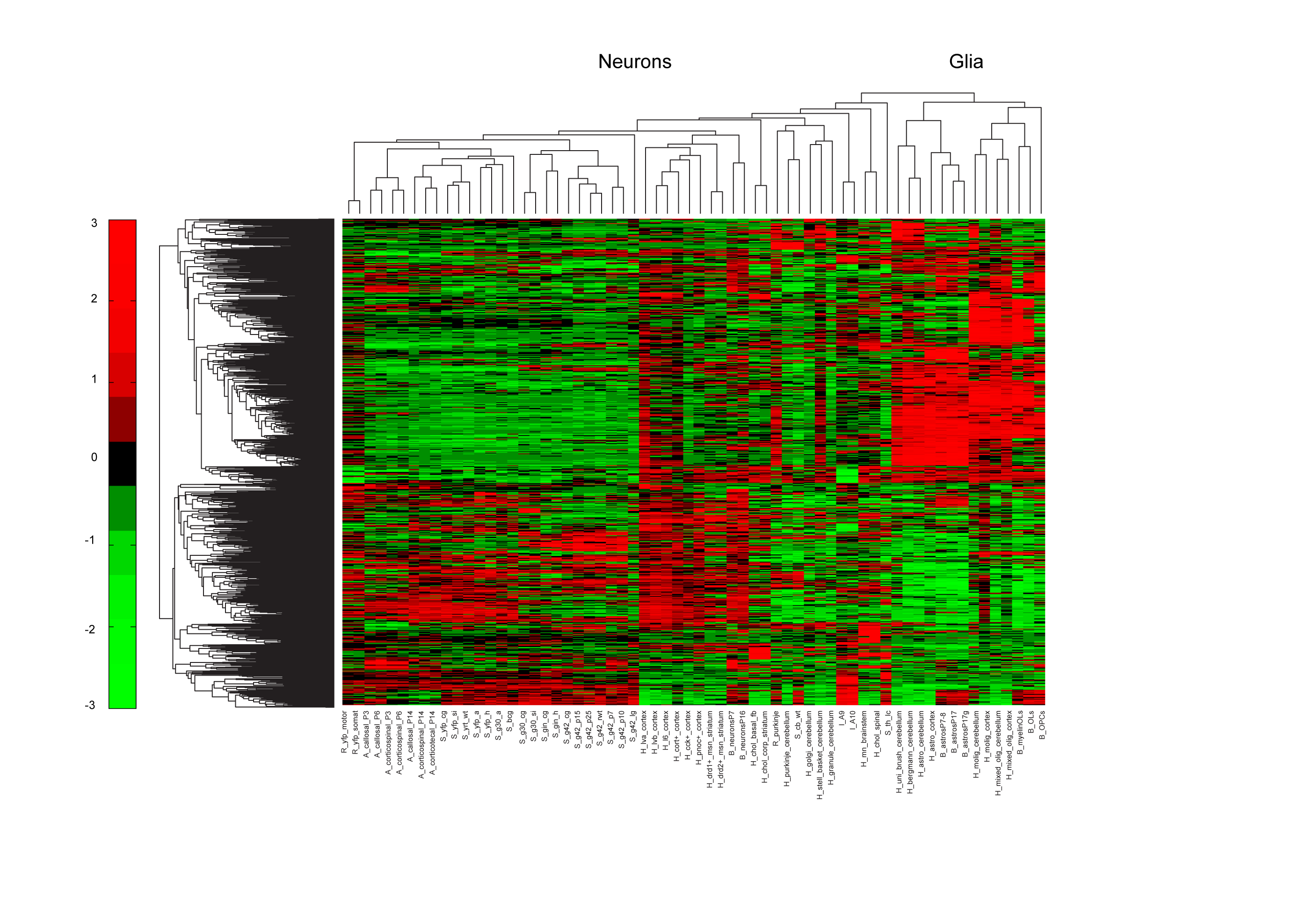

Supplement: Figure S2 — Dendrograms and heat map of highly significantly differentially expressed genes (see Methods ). Genes (rows) and cell types (columns) were clustered using Euclidean distance metric and average linkage. Microarray signal intensity values were standardized (across rows) such that the mean (i.e. mean signal value of a given gene across all samples) is zero and the standard deviation is one. Notice the primary division of glia and neurons. (TIF) [file pone.0016493.s002.tif]

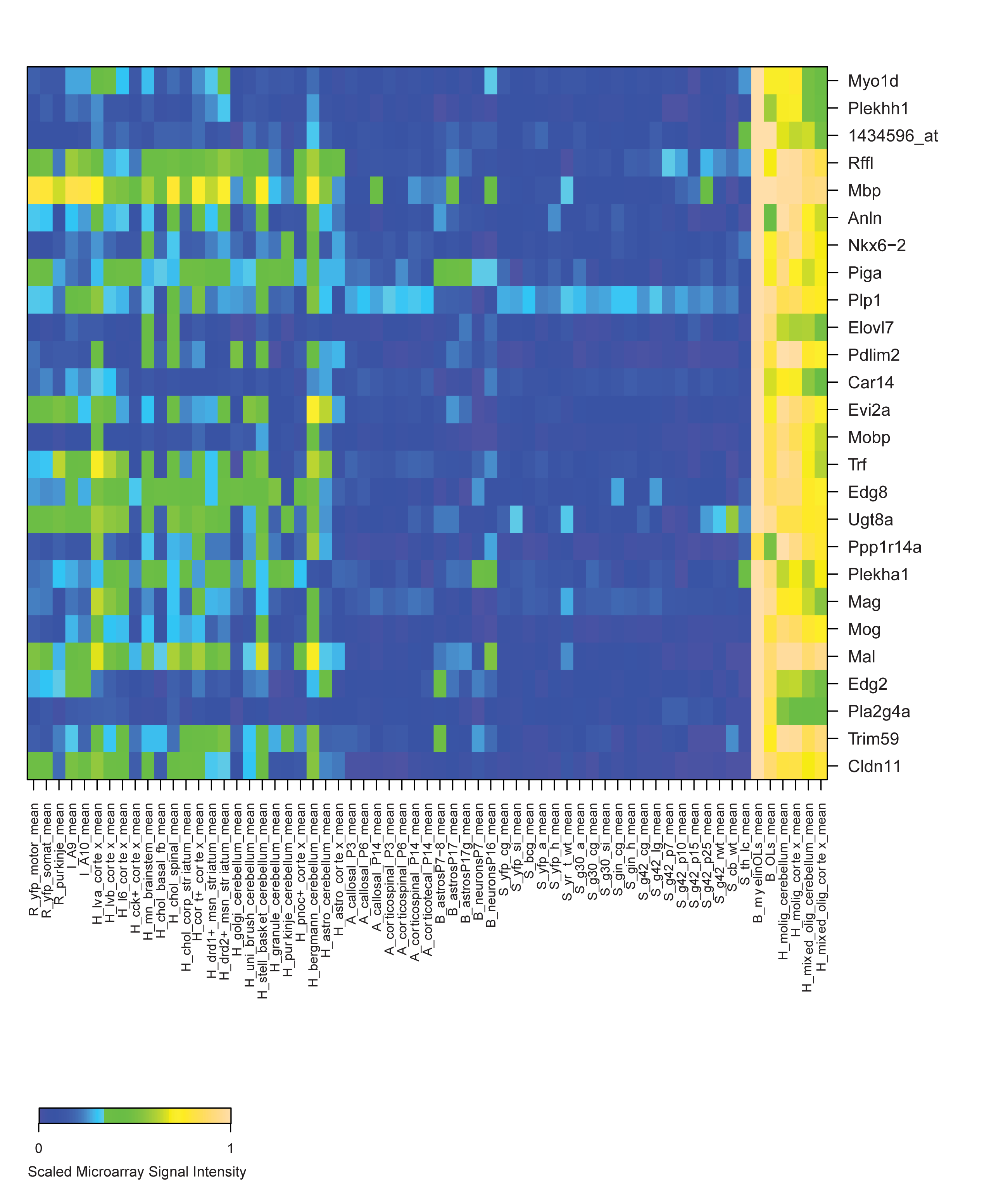

Supplement: Figure S3 — Normalized signal intensities of astrocyte enriched genes selected by clustering. Columns are sorted by method. (TIF) [file pone.0016493.s003.tif]

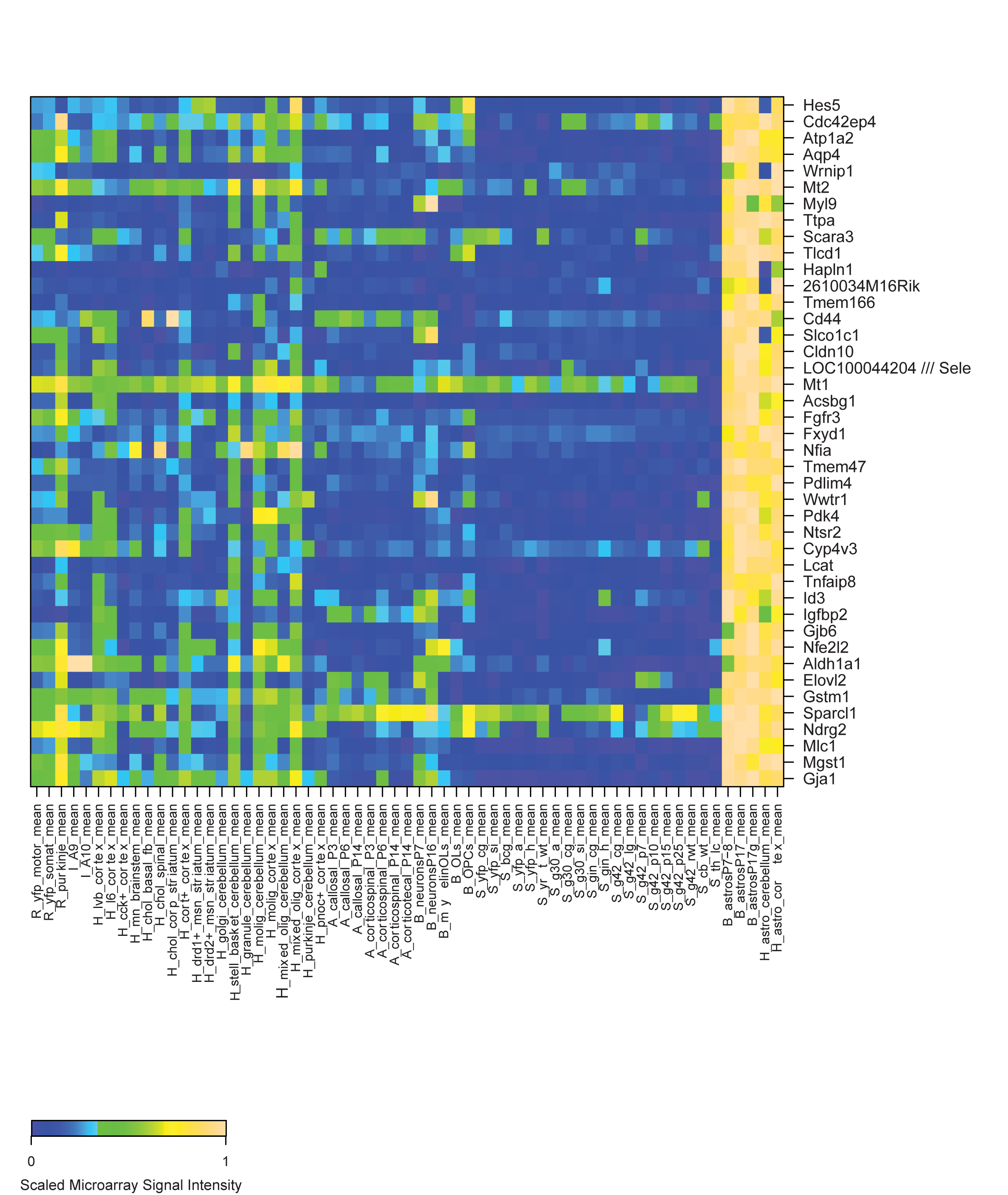

Supplement: Figure S4 — Normalized signal intensities of oligodendrocyte enriched genes selected by clustering. Columns are sorted by method. (TIF) [file pone.0016493.s004.tif]

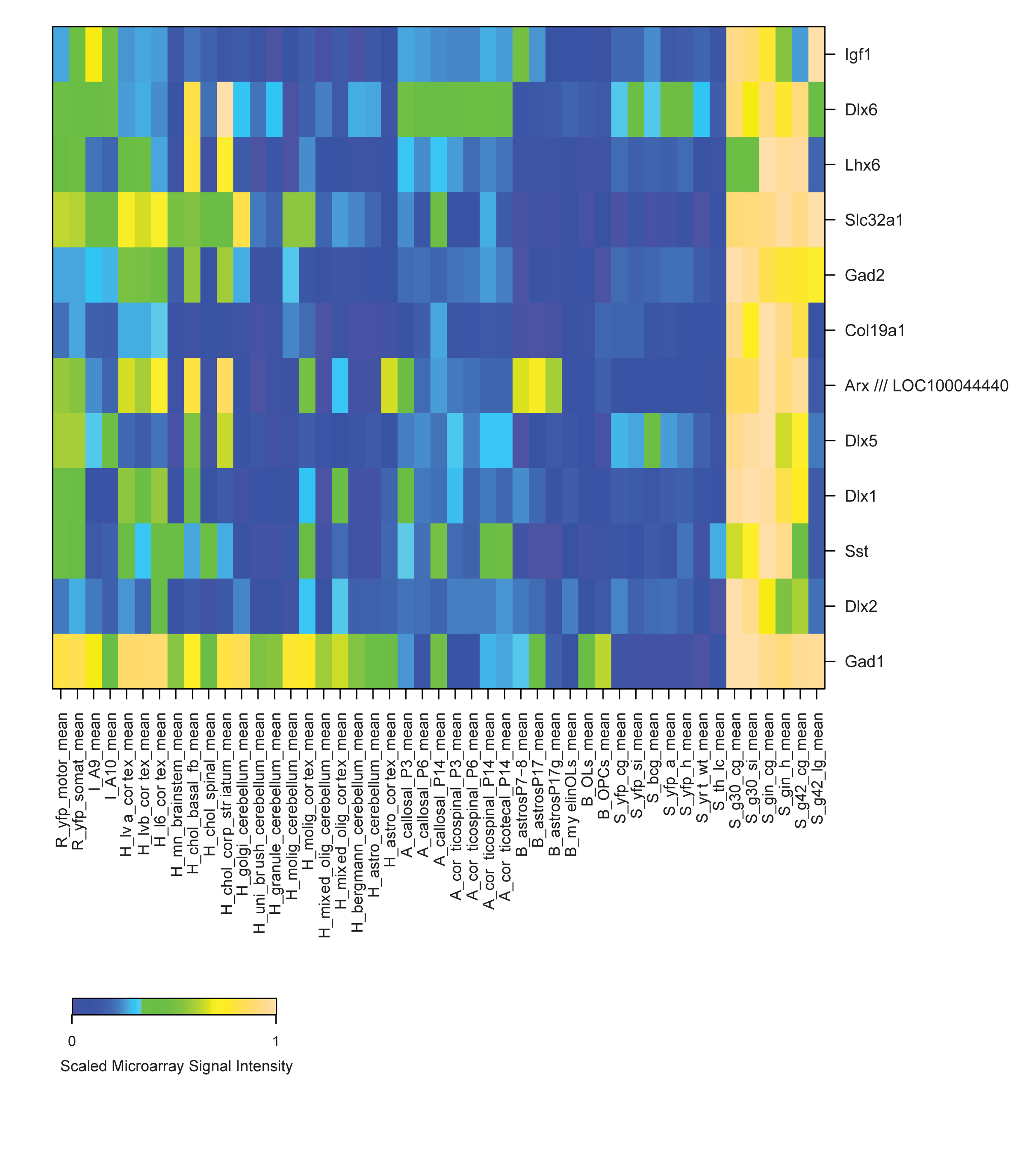

Supplement: Figure S5 — Normalized signal intensities of GABAergic neuron enriched genes selected by clustering. Columns are sorted by method. (TIF) [file pone.0016493.s005.tif]

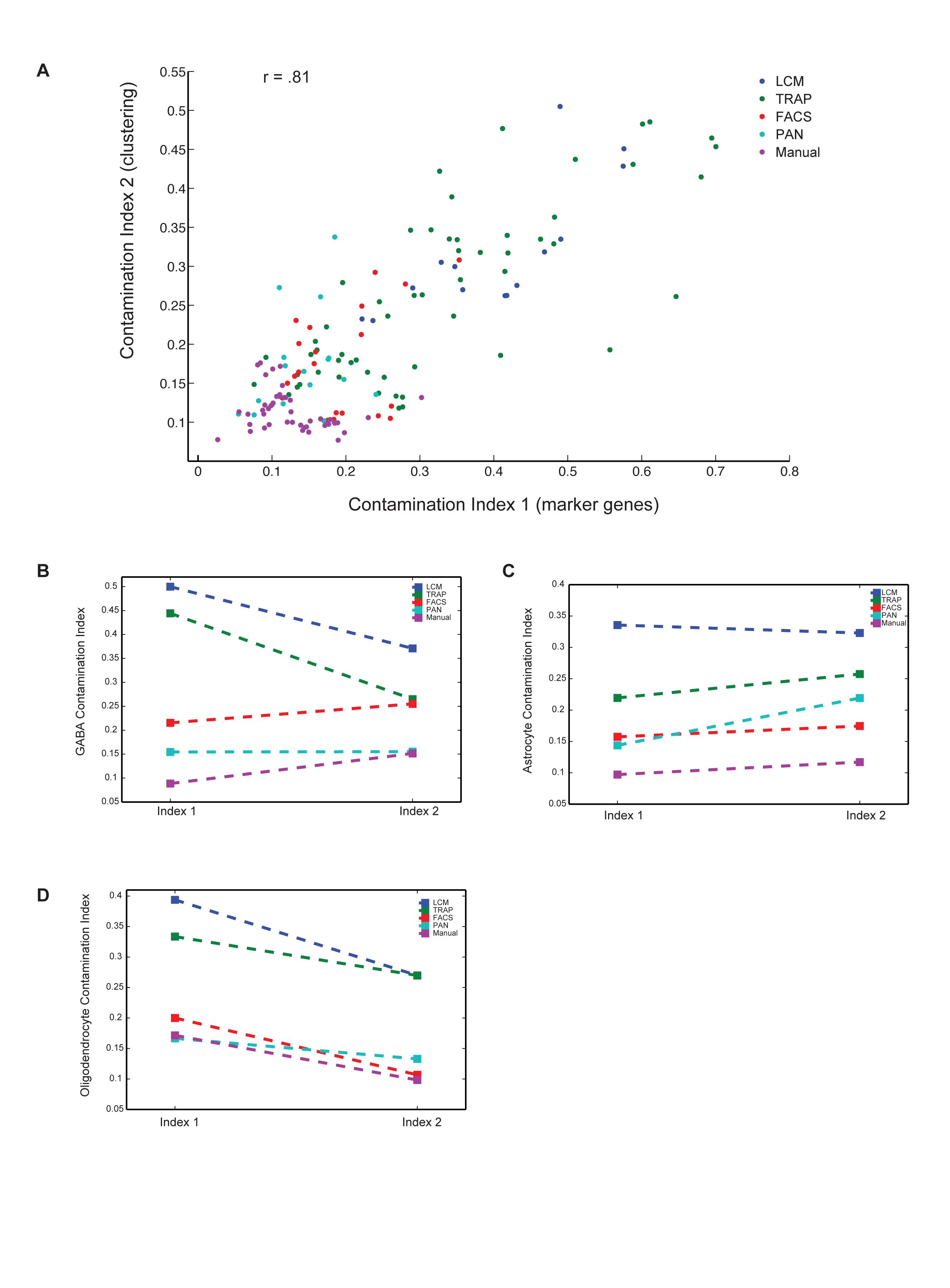

Supplement: Figure S6 — Comparison of two different contamination indices. The first was calculated based on the expression of well established marker genes alone, whereas the second was based on the expanded sets of genes selected by clustering (Figures S3, S4, S5). (A) Scatter plot of the two different contamination indices (correlation = .81). Comparison of the mean contamination indices for each method for (B) GABA contamination, (C) astrocyte contamination, and (D) oligodendrocyte contamination. (TIF) [file pone.0016493.s006.tif]

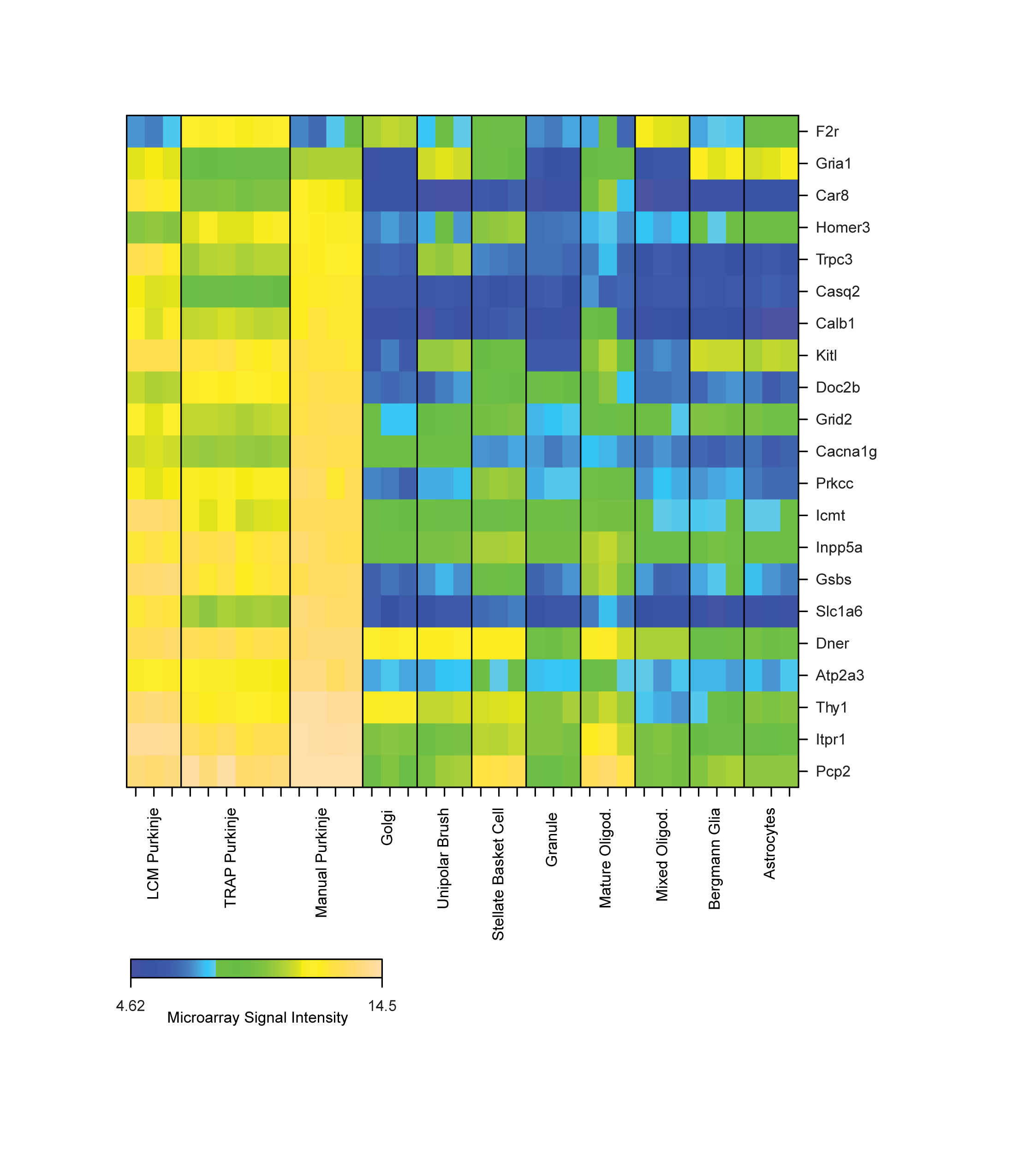

Supplement: Figure S7 — Purkinje samples from all three methods identify most known marker genes. Microarray signal levels are represented as a heat map for all cerebellar samples. (TIF) [file pone.0016493.s007.tif]

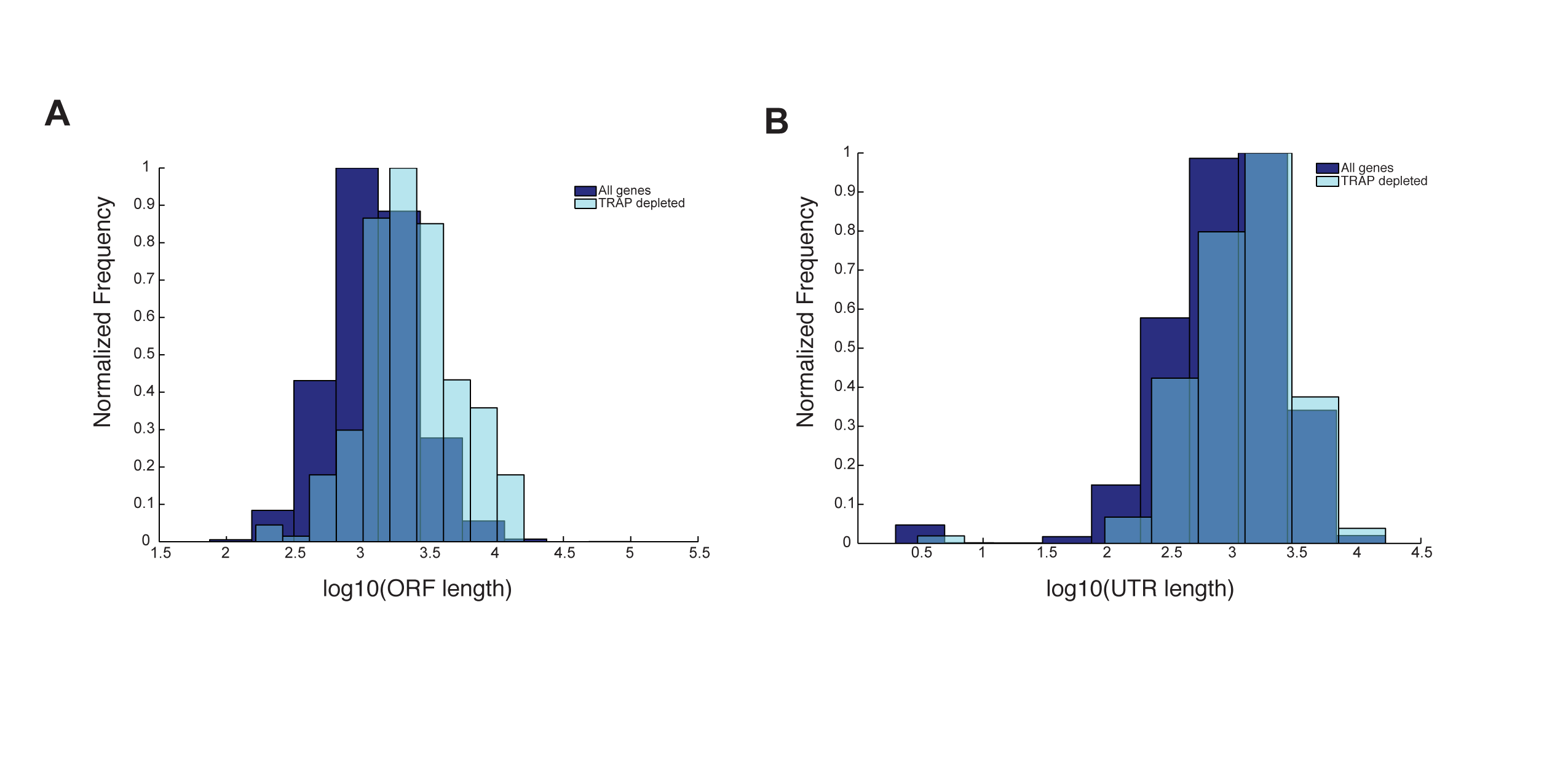

Supplement: Figure S8 — The mean UTR and ORF lengths of TRAP depleted genes are significantly higher than the mean UTR and ORF lengths for all annotated genes on the MOE 430 A gene chip, and show modest but significant correlation with the degree of suppressed expression. (A) Histogram depicting the normalized frequency of ORF lengths for all genes and for only TRAP depleted. (B) Histogram depicting the normalized frequency of UTR lengths for all genes and for only TRAP depleted. (TIF) [file pone.0016493.s008.tif]

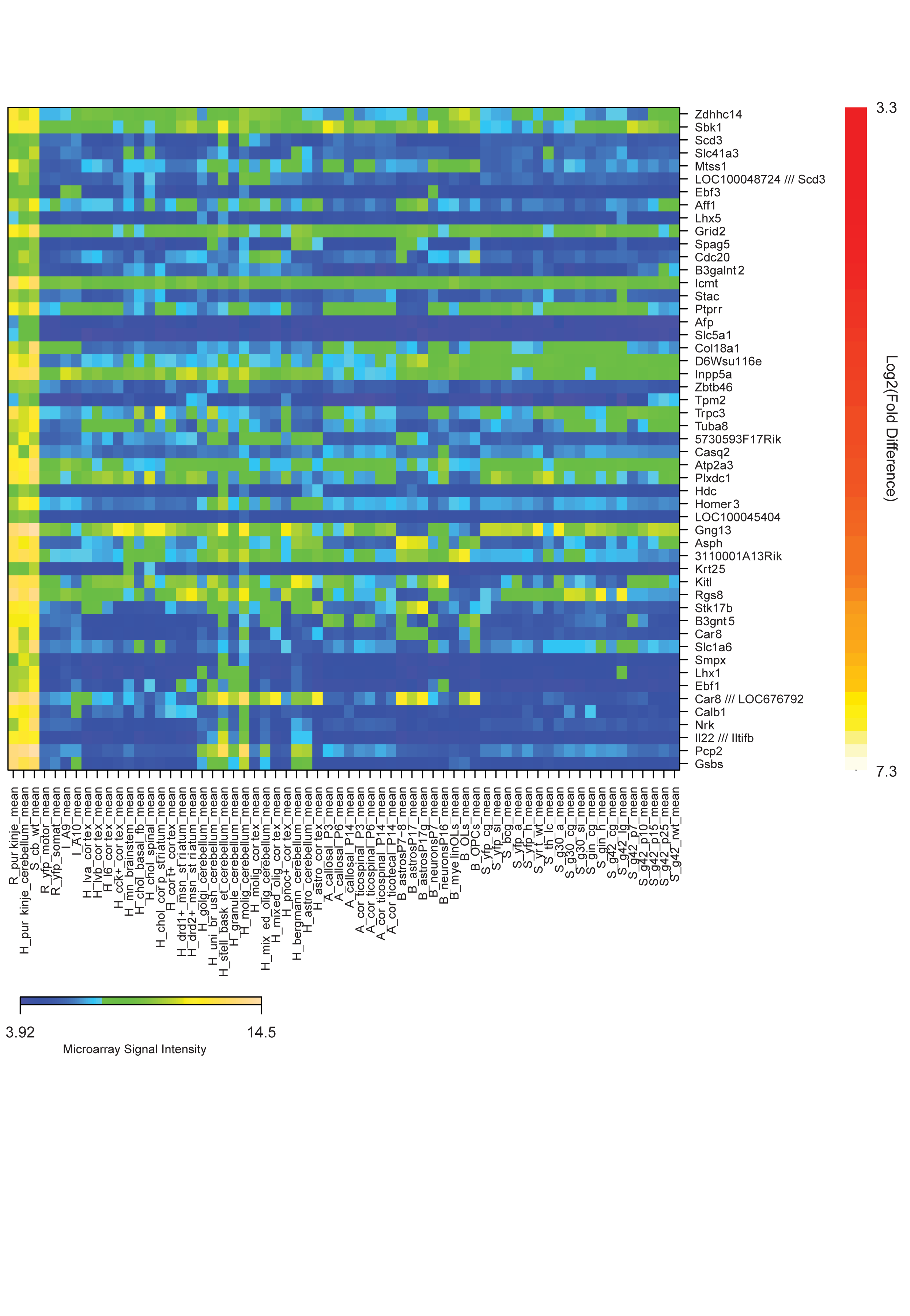

Supplement: Figure S9 — Heat map of purkinje enriched genes. (TIF) [file pone.0016493.s009.tif]
